# Gross anatomy of coronary veins of the European bison (*Bison bonasus*)

**DOI:** 10.1186/s12917-020-2259-0

**Published:** 2020-02-03

**Authors:** Karolina Barszcz, Michał Polguj, Karolina Goździewska-Harłajczuk, Joanna Klećkowska-Nawrot, Katarzyna Olbrych, Robert Haładaj, Marta Kupczyńska

**Affiliations:** 1 Department of Morphological Sciences, Institute of Veterinary Medicine, Warsaw University of Life Sciences – SGGW, Nowoursynowska 159, 02-776 Warsaw, Poland; 2 Department of Normal and Clinical Anatomy, Chair of Anatomy and Histology, Medical University of Lodz, Żeligowskiego 7/9, 90-752 Lodz, Poland; 3 Department of Animal Physiology and Biostructure, Faculty of Veterinary Medicine, University of Environmental and Life Sciences, Kożuchowska 1/3, 51-631, Wroclaw, Poland

**Keywords:** Great cardiac vein, Middle cardiac vein, Right cardiac veins, Heart vascularization, European bison

## Abstract

**Background:**

Although significant efforts have been put into restituting the European bison (*Bison bonasus*) and increasing its population, it remains a globally endangered species and requires conservation. Protection programs of bison indicate the need for morphological studies of their individual systems, in order to enhance restitution programs and enable appropriate veterinary care. The aim of this study was to investigate the morphology of the coronary veins of the European bison (*Bison bonasus*).

**Results:**

The study was carried out on 78 hearts of European bison of both sexes, aged 5 to 21 years. The subepicardial veins were clearly visible after being filled with dyed synthetic latex (LBS 3060), Plastogen G and Batson’s No. 17. In all the studied animals, the great cardiac vein drains into the coronary sinus. The interventricular paraconal branch and the circumflex branch of the great cardiac vein were distinguished. The left marginal vein, which originated in the apical area or halfway along the length of the left ventricular margin, was easily identified in 65 animals (83%). In most animals (51 bison, 65%), the middle cardiac vein drained into the coronary sinus, while in some animals it drained into the right atrium (27 animals, 35%).

**Conclusions:**

Blood from the cardiac walls is drained into the great cardiac vein, the middle cardiac vein, the right coronary veins and numerous small cardiac veins.

## Background

Coronary circulation has been studied in various vertebrate classes, such as mammals [1–12], birds [13], reptiles [14] and fish [15]. However, few reports focus on the cardiac coronary venous anatomy. The coronary venous morphology has been reported in the domestic cat (*Felis silvestris F. catus*), [16, 17], Angora goat (*Capra hircus*), Akkaraman sheep (*Ovis aries*) [18], Tuj sheep [19], porcupine (*Hystrix cristata*) [20], ringed seal (*Phoca hispida*) [21], Angora rabbit (*Oryctolagus cuniculus*) [22], New Zeland wite rabbit [23, 24] and the Wistar rat (*Rattus norvegicus domesticus*) [25]. Nonetheless, the available literature provides no details on the topography of coronary veins and their ramifications in the European bison.

Blood from the cardiac walls is drained into the great cardiac vein, the middle cardiac vein, the right coronary veins and numerous small cardiac veins. Similarly to the lymphatic system, the venous system is characterised by variable vessel anatomy. This general biological finding also applies to the venous drainage of the cardiac walls. Hence, it may be difficult to identify individual veins and their ramifications.

The aim of this study was therefore to investigate the morphology of the cardiac venous system in the European bison (*Bison bonasus*).

## Results

### Morphology of the vena cordis magna

**Fig. 1:**
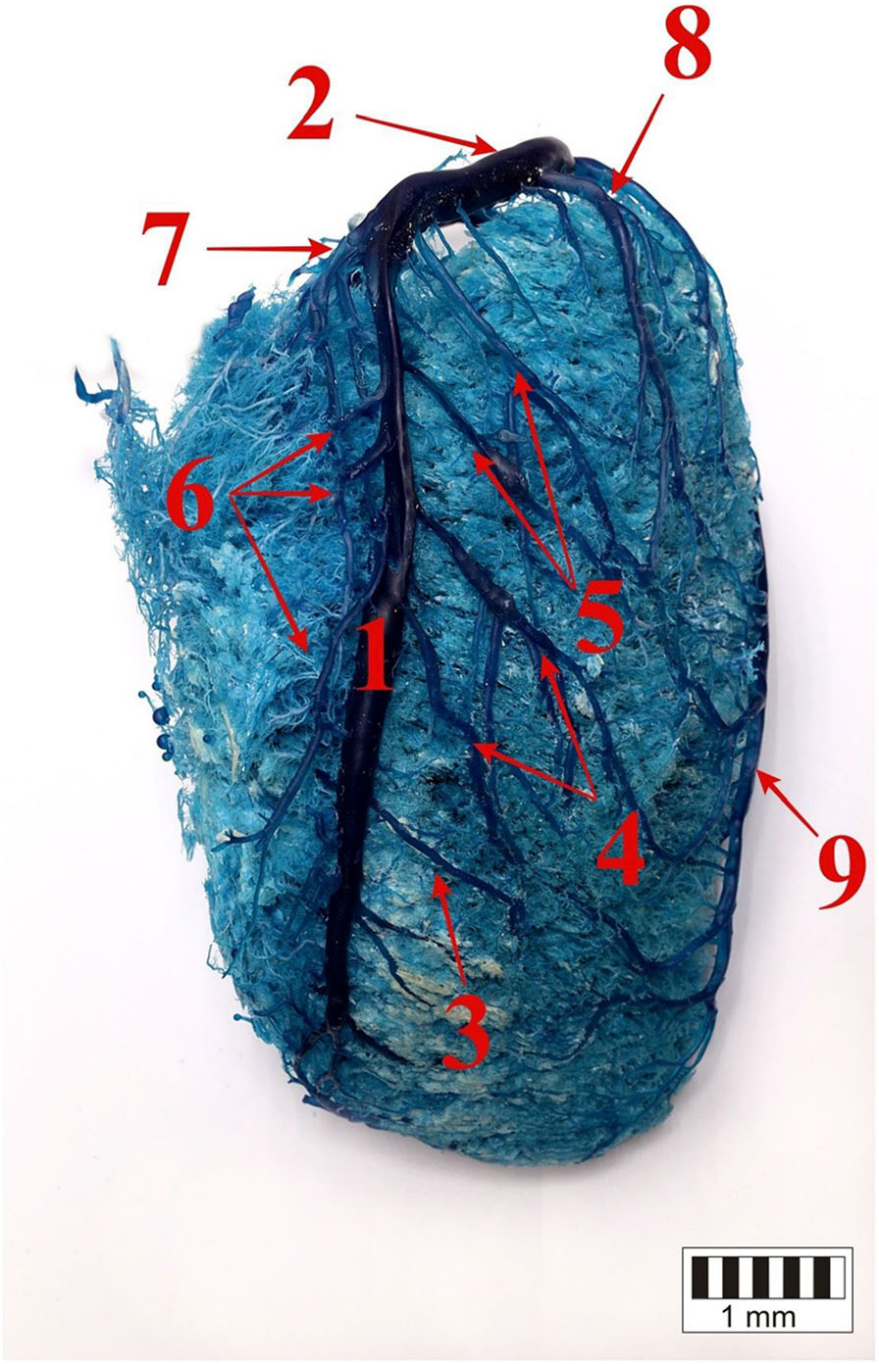
The auricular surface of the heart. 1 – the paraconal interventricular branch of the great cardiac vein, 2 – the circumflex branch of the great cardiac vein, 3 – the distal collateral vein, 4 – the intermediate collateral vein, 5 – the proximal collateral vein, 6 – the collateral veins of the right ventricle, 7 – the left vein of the atrial cone, 8 – proximal vein of the left ventricle, 9 – the left marginal vein

**Fig. 2:**
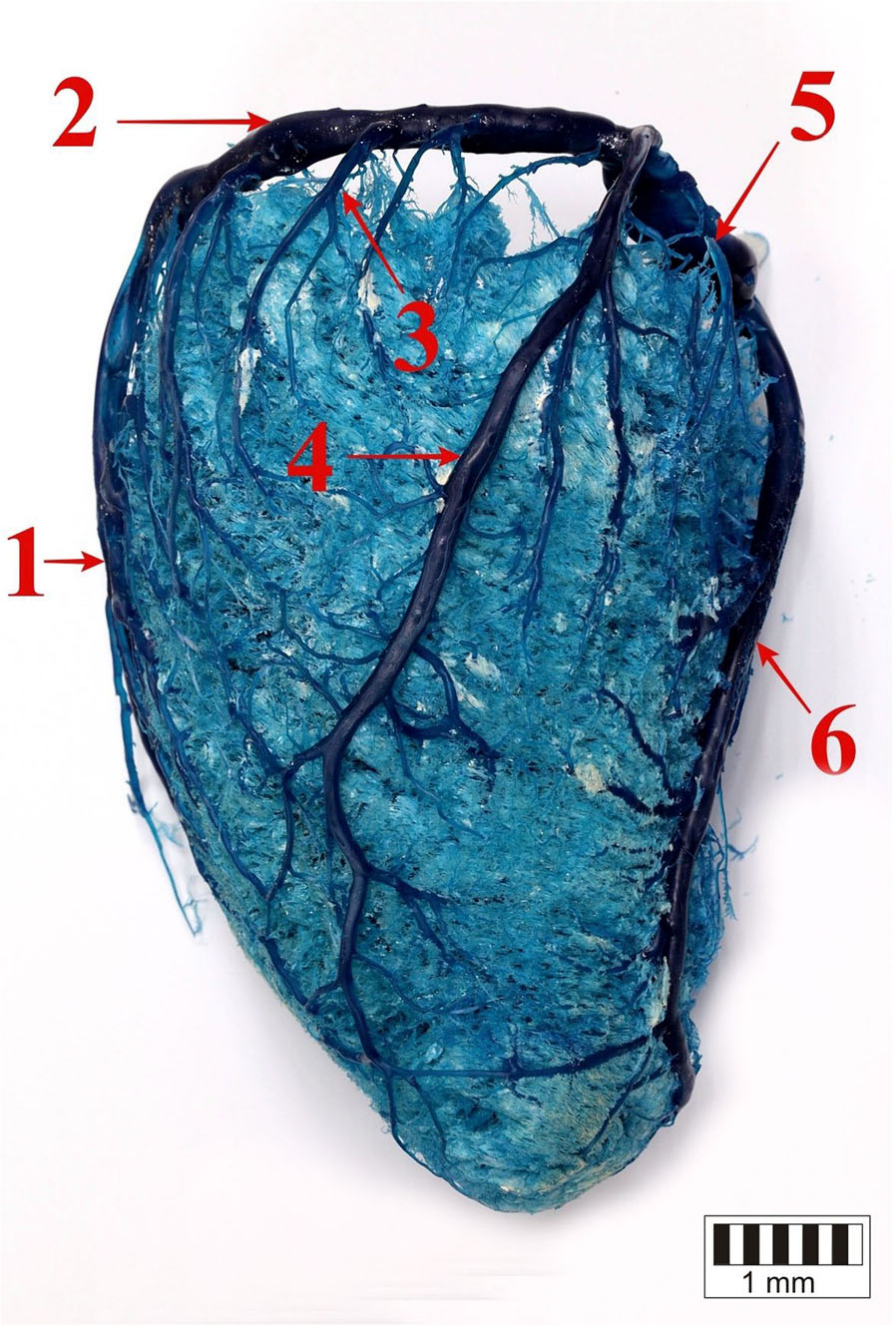
The left ventricular border of the heart. 1 – the paraconal branch of the great cardiac vein, 2 – the circumflex branch of the great cardiac vein, 3 – the proximal vein of the left ventricle, 4 – the left marginal vein, 5 – the distal vein of the left ventricle, 6 – the middle cardiac vein

**Fig. 3:**
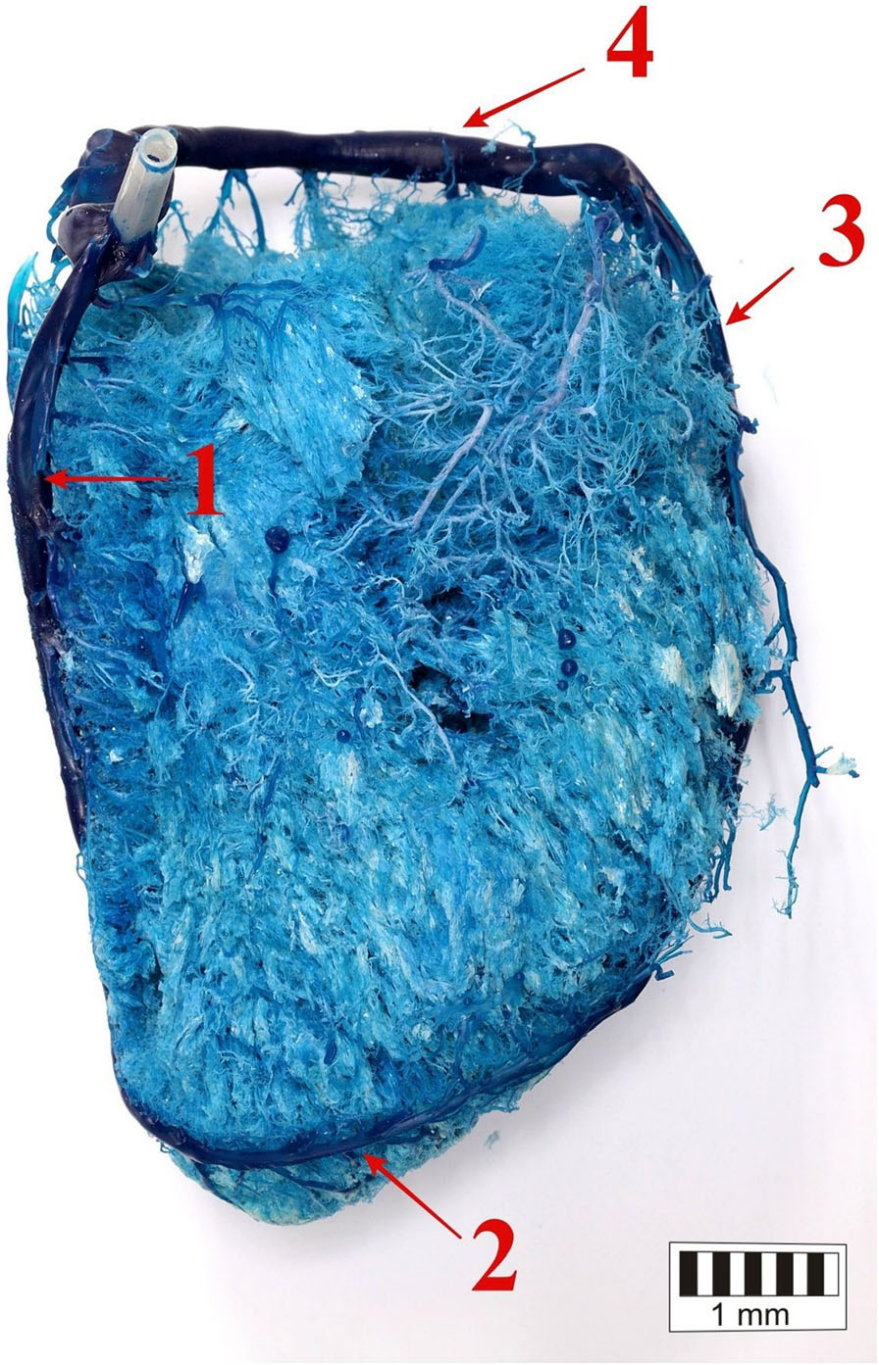
The right ventricular border of the heart. 1 – the middle cardiac vein (the atrial surface of the heart), 2 – the middle cardiac vein (the auricular surface of the heart), 3 – the paraconal branch of the great cardiac vein, 4 – the circumflex branch of the great cardiac vein

The presence of the great cardiac vein (*v. cordis magna*), which formed the coronary sinus, was confirmed in all the studied bison. It divided into the interventricular paraconal branch and the circumflex branch (Figs. 1, 2, 3).

**Fig. 4:**
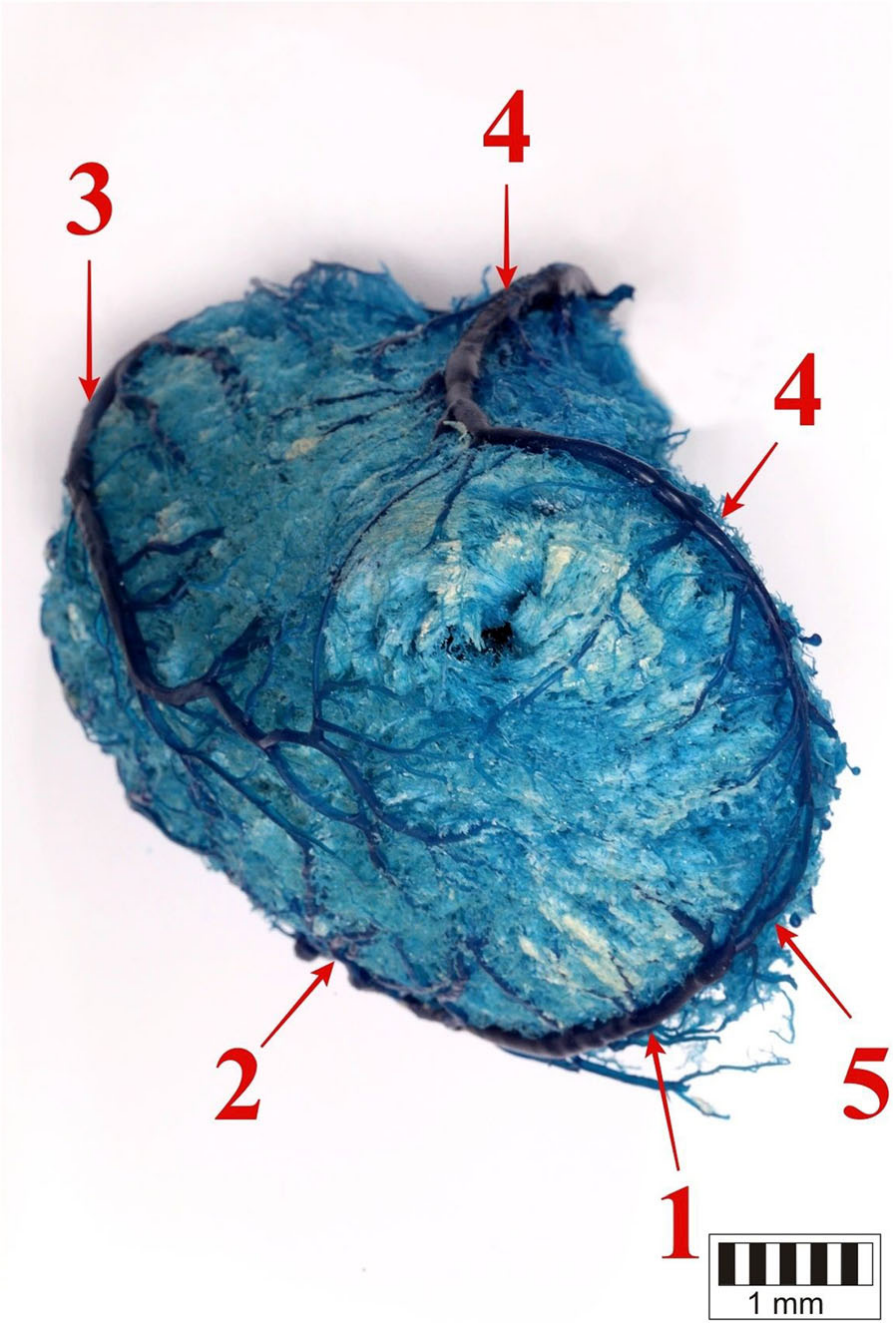
The cardiac apex. 1 – the paraconal branch of the great cardiac vein (the atrial surface of the heart), 2 – the paraconal branch of the great cardiac vein (the auricular surface of the heart), 3 – the left marginal vein, 4 – the middle cardiac vein, 5 – anastomoses between the great and middle cardiac vein

**Fig. 5:**
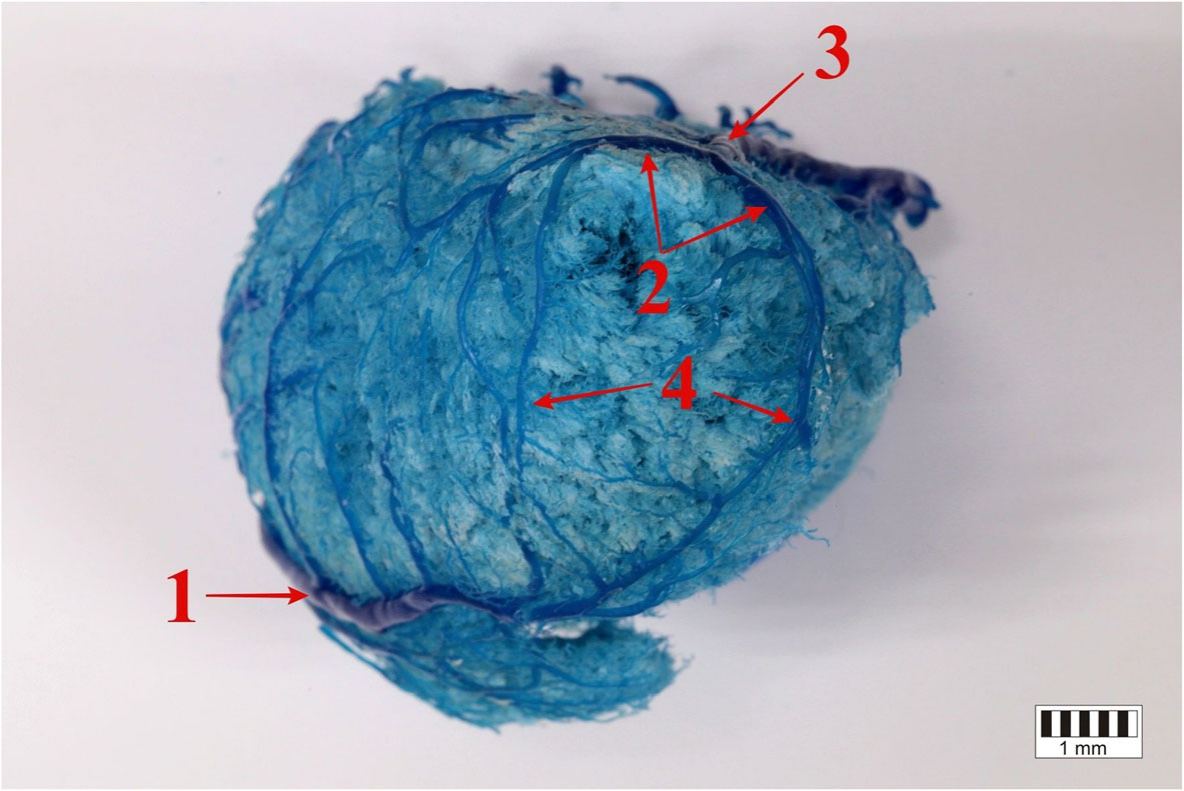
The cardiac apex. 1 – the paraconal branch of the great cardiac vein (the auricular surface of the heart), 2 – middle cardiac vein (two branches on the auricular surface of the heart), 3 – the middle cardiac vein (the atrial surface of the heart), 4 – anastomoses between great and middle cardiac vein

The interventricular paraconal branch arose from two thin trunks on the auricular surface, above the notch of the cardiac apex (Figs. 4, 5). Both trunks merged into one branch running in the paraconal interventricular groove.

Side tributaries that collected blood from both ventricular walls drained into the trunk of the interventricular paraconal branch of the great cardiac vein. Those included the *v. collateralis distalis ventriculi sinistri*, *v. collateralis intermedia ventriculi sinistri*, *v. septi interventriculorum*, *v. coni arteriosi sinistra* and the *vv. collaterales proximales ventriculi sinistri*. The collateral veins ran in an oblique course on the cardiac muscle directly under the epicardium with individual variability in their number. The veins draining blood from the left ventricle were larger and extended from the left ventricular border. Those that arose on the right ventricular wall were of varying width. All the branches arose from single small tributaries forming variable, rich vascular patterns. Numerous connections were observed between branches originating from the same and neighbouring collateral veins (Fig. 1).

The distal collateral vein arose in close proximity to the right ventricular border above the notch of the cardiac apex and drained into the interventricular paraconal branch of the great cardiac vein (Fig. 1).

The intermediate collateral vein ran from the left ventricular wall, close to the left ventricular border into the great cardiac vein mid-way along the length of the ventricles (Fig. 1). This vessel was poorly developed in 17 animals (22%), and it collected blood only from the area of the paraconal interventricular groove. In 14 bison (18%), two parallel collateral branches were found.

The vein of the interventricular septum formed from two small trunks, which later merged into a single branch and drained into the interventricular paraconal branch of the great cardiac vein.

The left vein of the arterial cone originated from small vessels on the arterial cone close to the right ventricular border and drained into the interventricular paraconal branch of the great cardiac vein above the vein of the interventricular septum (Fig. 1).

There were between one and four proximal collateral veins, the most developed vessels, which drained into the interventricular branch of the great cardiac vein (Fig. 1). They emerged from the left ventricular border and drained into the distal section of the interventricular branch of the great cardiac vein. They formed numerous anastomoses with tributaries of the left marginal vein, the left accessory marginal vein and the middle cardiac vein. In 20 cases (26%), one of the proximal collateral veins was of a considerable size and ran from the apex of the heart. In those animals, the left accessory marginal vein was shortened.

In all the studied bison, the circumflex branch of the great cardiac vein was a direct extension of the interventricular paraconal branch of the great cardiac vein (Figs. 1, 2, 3). The described vessel ran in the coronary groove on the auricular surface, under the left auricle. Then, the circumflex branch of the great cardiac vein passed along the left ventricular border and ran on the atrial surface. In all the animals, it emptied into the coronary sinus in the right atrium. It was surrounded by a thick layer of adipose tissue.

Veins that emptied into the circumflex branch of the great cardiac vein included the *v. proximalis ventriculi sinistri*, *v. marginis ventricularis sinistri accessoria*, *v. marginis ventricularis sinistri* and the *v. distalis ventriculi sinistri*.

The proximal vein of the left ventricle was poorly developed. It ran from the auricular surface, below the coronary groove and was the first vessel to drain into the circumflex branch of the great cardiac vein (Fig. 2).

The accessory left marginal vein emerged halfway along the length of the left ventricular margin, ran across the auricular surface and drained into the circumflex branch of the great cardiac vein under the left auricle. In 21 animals (27%), it was of considerable size, ran from the apex of the heart along the entire length of the left ventricular border and drained blood from the wall of the left ventricle. In those animals, the left marginal vein was less developed. In one bison (2%), the described vessel formed two parallel trunks.

The development of the left marginal vein varied between individuals. In 40 bison (51%), it was well developed and ran from the apex of the heart (Figs. 2, 4). It received several tributaries from the auricular and atrial surface (branches of the middle cardiac vein and the interventricular paraconal branch of the great cardiac vein). In 25 (32%) of the animals, the left marginal vein was poorly developed and emerged halfway along the left ventricular border. In the remaining 13 (17%) bison, it was represented by a single small vessel that collected blood from a small area close to the coronary groove. Due to the poor development of the the left marginal vein, tributaries of the interventricular paraconal branches of the great cardiac vein as well as the middle cardiac vein and the accessory left marginal vein were noticeably larger in 38 bison.

The distal collateral branch of the left ventricle emerged at the level of the ventricles, ran along the atrial surface and drained into the distal segment of the circumflex branch of the great cardiac vein or directly into the coronary sinus (Fig. 2).

Varying numbers of more or less prominent vessels draining blood from the left atrial wall, namely the *v. proximalis atrii sinistri, V. intermedia atrii sinistri* and the *v. distalis atrii sinistri* drained into the circumflex branch of the great cardiac vein. Those vessels ran along the outer surface of the medial wall of the left auricle. There were also small tributaries running between the three main trunks.

### Morphology of the v. cordis media

**Fig. 6:**
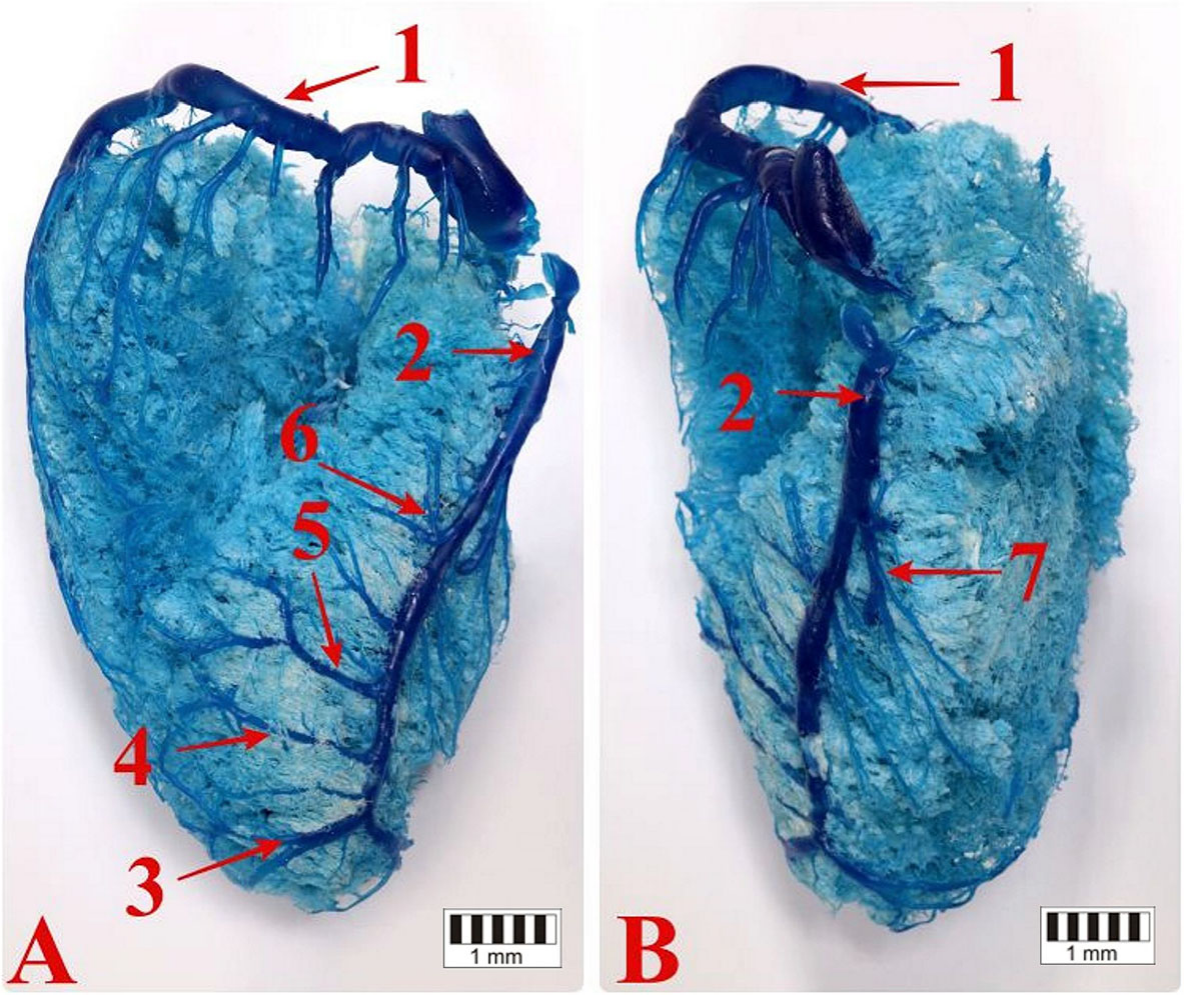
**a** – The atrial surface of the heart and the collateral veins of the left ventricle. **b** – The atrial surface of the heart and the collateral veins of the right ventricle. 1 – the circumflex branch of the great cardiac vein, 2 – the middle cardiac vein, 3 – the apical vein, 4 – the distal collateral vein, 5 – the intermediate collateral vein, 6 – the proximal collateral vein, 7 – the oblique vein of the right ventricle

The middle cardiac vein was a well-developed vessel. It emerged in the form of two branches on the auricular surface of the distal third of the interventricular paraconal groove. The branches formed connections with the veins of the great cardiac vein and crossed the notch of the cardiac apex (Figs. 4, 5). They then ran along the atrial surface and in the interventricular subsinousal groove. In most cases (51 bison, 65%), the middle cardiac vein drained into the coronary sinus, while in some cases it drained into the right atrium (27 bison, 35%). The initial segment of the vessel collected blood from small tributaries on the auricular surface of both ventricles, while the following vessels drained into the segment running in the subsinuosal interventricular groove: *v. apicis cordis*, *r. collateralis distalis*, *r. collateralis intermedia*, *r. collateralis proximalis*, *v. obliqua ventriculi dextri* (Fig. 6).

The apical vein emerged from the apex of the heart on the left ventricular border and drained into the corresponding vein in the notch of the apex of the heart (Fig. 6a).

The distal collateral branch originated as small vessels on the left ventricular border and entered the middle cardiac vein above the notch of the apex. In five bison (6%), it formed a common trunk with the intermediate collateral branch (Fig. 6a).

The intermediate collateral branch originated halfway along the length of the left ventricular border and drained into the middle cardiac vein halfway along the length of the subsinousal interventricular groove (Fig. 6a).

The proximal collateral branch originated from 2 to 5 small vessels and collected blood from the left ventricle located under the coronary sinus (Fig. 6a).

The oblique vein of the right ventricle originated at the halfway point of the right ventricular border. It ran obliquely on the atrial surface and drained into the distal section of the middle cardiac vein (Fig. 6b).

### Right cardiac veins

There were 4–6 cardiac veins, which collected blood from the conus arteriosus and the right atrium. These included the *v. semicircumflexa coni arteriosi*, *v. distalis ventriculi dextri* and the *vv. cordis dextri accessoriae*.

The semicircumflex vein of the atrial cone originated on the auricular surface below the pulmonary trunk and drained into the right atrium under the right auricle. The following veins drained into it along its course: *vv. coni arteriosi dextrae*, *v. proximalis ventriculi dextri*, *v. marginis ventricularis dextri*. Two to three right veins of the atrial cone drained blood from the atrial cone. The proximal vein of the right ventricle collected blood from the right ventricle in close proximity to the right ventricular border and drained into the semicircumflex vein of the atrial cone. The right marginal vein originated in the right ventricular border and drained into the distal segment of the semicircumflex vein of the atrial cone. In 10 animals (13%), it drained directly into the right atrium.

The distal vein of the right ventricle ran along the atrial surface and drained into the right atrium.

There were 2–4 accessory right cardiac veins. They collected blood from the wall of the right ventricle on the atrial surface and drained into the right atrium. In five cases (6%), all the accessory right cardiac veins and the distal vein of the right ventricle formed a common trunk that ran in the coronary sulcus under the right auricle and drained into the right atrium next to the coronary sinus ostium.

## Discussion

Available studies report two parts of the great cardiac vein – the interventricular paraconal branch and circumfex branch. A dychotomous great cardiac vein has been reported in ruminants, such as the cattle [3], goat [26], Tuj sheep [19], Angora goat as well as the Akkaraman sheep [18].

In the studied bison, the interventricular paraconal branch of the great cardiac vein originated from two thin trunks on the auricular surface above the notch of the cardiac apex. According to Atalar et al. [20], the described vessel emerges at the cardiac apex in all domestic animals apart from the cat.

In the studied bison, numerous anastomoses were found between the interventricular paraconal branch of the great cardiac vein and the middle cardiac vein. Similar observations were made by Hegazi in other ruminant species [27] and by other researchers in the Akkaraman sheep, Angora goats [18], cattle [12] and Tuj sheep [19].

In the bison in this study, individually variable numbers of collateral branches ran obliquely, directly under the epicardium. Vessels draining blood from the left ventricular wall were significantly larger and ran from the left ventricular border. Similar observations were made in the goat [26]. In the studied bison, proximal collateral branches were the most developed vessels and drained into the paraconal interventricular branch of the great cardiac vein. There were from one to four vessels observed in each animal. They emerged at the left ventricular border and drained into the distal segment of the paraconal interventricular branch of the great cardiac vein.

In all the bison, the circumflex branch of the great cardiac vein was a direct extension of the interventricular paraconal branch of the great cardiac vein. The described vessel ran in the coronary groove on the auricular surface and then passed along the left ventricular border and ran on the atrial surface.

In the bison, the left marginal vein was developed to varying degrees. In the majority of the animals (65 bison, 83%), it was well developed and originated at the apex of the heart or mid-way along the left ventricular border. The left marginal vein received a series of tributaries from the auricular and atrial surfaces. According to literature the left marginal vein drained into the coronary sinus in Akkaraman sheep and into the great cardiac vein in Angora goats [18].

In all the studied bison, the great cardiac vein drained into the coronary sinus, as in the Angora goat, Akkaraman sheep [18] and Tuj sheep [19].

The middle cardiac vein was a well-developed vessel. It most commonly originated from two branches on the auricular surface in the distal third of the interventricular paraconal groove. These branches connected with venous ramifications of the great cardiac vein. They then ran along the atrial surface and in the interventricular subsinousoal groove. A similar course of these vessels was reported in the Tuj sheep [19].

In 65% of the studied bison, the middle cardiac vein drained into the coronary sinus and in 35% of the animals into the right atrium. Many authors reported that the middle cardiac vein opened into the coronary sinus in domestic species [6, 16, 27–29].

The right cardiac veins vary depending on species and individuals. They originate at various levels on the atrial surface and the auricular surface of the right ventricular wall and on the right ventricular border. They then receive numerous small tributaries that form a wide vascular network. The variations in the subepicardial capillary system impede the identification of individual vessels, which is thus based on their topographical localisation. In the studied bison, there were between four and six right cardiac veins that collected blood from the arterial cone and the right ventricle.

In all the studied bison, the right cardiac veins drained independently into the right atrium. According to literature, this is the most commonly encountered drainage system. There have also been reports of these vessels draining into the right ventricle, as in Thuj sheep [19], cattle [10], Angora goats or Akkaraman sheep [18].

The European bison is an endangered species. National and breeding centers have been replenished with bison from Bialowieza, significantly contributing to the population growth of this species. However, this species still requires human protection. Currently, numerous breeding, as well as in situ and ex situ study programs are being implemented [30]. Protection programs of bison indicate the need for morphological studies of their individual systems, in order to enhance restitution programs and enable appropriate veterinary care.

## Conclusions

Blood from the cardiac walls is drained into the great cardiac vein, the middle cardiac vein, right cardiac veins and numerous small tributaries. In all the studied animals, the great cardiac vein drained into the coronary sinus. The interventricular paraconal branch and the circumflex branch of the great cardiac vein were identified. In most animals, the left marginal vein was well developed. It originated at the apex or mid-way along the left ventricular border. The middle cardiac vein drained into the coronary sinus, or, less commonly, into the right atrium.

## Methods

The study was conducted on 78 hearts of European bison of both sexes that were from five to 21 years old.

The Bialowieza National Park culled the European bison for reasons other than for the purpose of this study, which included population control, bone fractures and car accidents. The permission for culling was issued by the Ministry of Environment and the General Director for the Environmental Protection in Poland (decision number: DOP-OZGIZ. 6401.06.7.2012.ls, DOP-OZ.6401.06.7.2012.ls.1 and DLP-III-4102-459/36490/14/ZK).

The hearts were collected by veterinary professionals during dissection, whereby a pathological examination of the whole body of the animals was performed. The autopsy protocols are available in the Bialowieza National Park. According to the Polish law, studies on tissues obtained *post-mortem* do not require an approval of the Ethics Committee) [31].

The pericardial sac and surrounding tissue were removed in order to obtain access to the coronary veins. Seventy hearts were dyed using synthetic LBS 3060 latex (Synthos Dwory Sp. z o.o, Poland) using a previously described method [2, 32–36] and were placed in 10% formalin for six weeks. The trunks of the coronary veins and their ramifications were then prepared.

The corrosion casts were obtained from eight hearts. First, a 0.9% NaCl solution was injected into the coronary veins to flush out clots. Next, 20 ml of a 3% glutaraldehyde solution in a pH 7.4 cacodylate buffer was injected into the coronary veins, which were then filled with colored Plastogen G (Plasto-Schmidt, Speyer, Germany) or Batson’s No. 17 (Polyscience, Incorporation, Warrington, US). The heart was then placed in water at 20 °C for 24 h in order to harden the resin. Following cast hardening, the specimen was placed in a 40% KOH solution at 50 °C for approximately 24 h to dissolve the organic tissue. Dissolved tissue residues were removed from the specimen through a 38 h continuous flushing with water. The specimen was cleaned using a fast wash with warm water and a small amount of standard washing liquid, followed by a final flush with distilled water. The cast was later dried using airflow at room temperature for two days. The method was successfully implemented in our previous studies [2, 32, 33, 36, 37].

Following the above described processing, the specimens were examined morphologically using a ECLERIS (HALOLUX 150) surgical microscope. The terminology used in the manuscript is in accordance with the prevailing veterinary nomenclature [38].

## Data Availability

Not applicable. The datasets used and/or analyzed during the current study are available from the first author - dr. Karolina Barszcz (karolina_barszcz@sggw.pl) on reasonable request.

## Abbreviations

*r.*: *Ramus*
*v.*: *Vena*
*vv.*: *Venae*
